# Extraction of Active Compounds from *Dioscorea quinqueloba* and Their Encapsulation Using Mucin and Chitosan for Application in Cosmetic Formulations

**DOI:** 10.3390/ma18102178

**Published:** 2025-05-08

**Authors:** Min Jae Shin

**Affiliations:** School of Life Science and Biotechnology, Gyeongkuk National University, Andong 36729, Republic of Korea; newminj@gmail.com

**Keywords:** *Dioscorea quinqueloba*, yam, microcapsule, mucin, chitosan, cosmetic formulation

## Abstract

The purpose of this study is to develop a fundamental material for cosmetics by encapsulating the extract obtained from *Dioscorea quinqueloba* using chitosan, a natural material. Active compounds were extracted using ethanol from *Dioscorea quinqueloba* produced in Andong, South Korea. These compounds were encapsulated in mucin extracted from *Dioscorea quinqueloba* and chitosan derived from cuttlefish bones to form microcapsules. The encapsulation process involves the formation of a W/O emulsion, followed by crosslinking with glutaraldehyde. The resulting microcapsules were examined by scanning electron microscopy, which revealed spherical structures with an average size ranging from 890 to 1130 nm. The toxicity, antioxidant activity, and anti-aging properties of these microcapsules were assessed to evaluate their potential use in cosmetic formulations. The microcapsules showed no toxicity at the concentrations used, and their antioxidant and anti-aging activities were significantly higher than those of the control group. These findings suggest that microcapsules have strong potential as components in cosmetic compositions.

## 1. Introduction

Yam belongs to the *Dioscoreaceae* family of the order *Liliales*, with over 650 species identified worldwide. It is widely distributed in Korea. Yams exhibit significant diversity through interspecies hybridization, resulting in a wide variety of root and leaf shapes. Based on the root shapes, yams can be classified as long, short, or round. Andong, where this study was conducted, is a major yam-producing region of Korea.

Yams are primarily consumed fresh but are also used as medicinal ingredients after proper drying. The main component of yam is starch; however, it also contains proteins, minerals, other nutrients, and medicinal compounds. Their medicinal components include saponins, choline, mucin, amylose, diosgenin, and arginine. Numerous pharmacological effects of these compounds have been reported [1,2,3,4,5,6]. For instance, arginine aids vascular endothelial cell formation [7] and platelet generation [8], whereas saponins are recognized for their anticancer [9] and anti-inflammatory properties [10].

Mucin is a mucilaginous substance widely distributed in nature, with glycoproteins as its main component. It is abundantly found in plants such as yam and lotus root, as well as in animals like snails and eels. Mucin plays a crucial role in protecting the gastric mucosa [11,12], promoting gut health [13,14], and helping to prevent constipation [15]. Additionally, it is gaining attention as a cosmetic ingredient due to its ability to keep the skin firm and aid in anti-aging [16,17,18].

Encapsulation technologies have become essential tools across multiple industries, such as pharmaceuticals, food, cosmetics, and materials science. These techniques enable efficient delivery, protection, and controlled release of active substances by enclosing them within a carrier matrix. Water-in-oil (W/O) emulsions stand out among various encapsulation methods because of their unique ability to encapsulate hydrophilic substances in hydrophobic environments [19,20,21,22,23,24]. A W/O emulsion consists of water droplets dispersed within a continuous oil phase stabilized by surfactants or emulsifiers. This structure is particularly effective for encapsulating water-soluble compounds such as proteins and peptides, ensuring their stability and protection in hydrophobic settings.

Chitin is found in the shells of crustaceans such as crabs and shrimp, the exoskeletons of insects, and the structural components of mollusks like squid [25,26]. Additionally, chitin is a major component of the cell walls of fungi and mushrooms [27]. Chitosan is a substance derived from chitin, produced by hydrolyzing the acetylamino group of chitin into an amine group. It is known to enhance the body’s natural healing ability and strengthen the immune system [28,29,30]. Furthermore, chitosan plays a role in absorbing and excreting cholesterol [31,32] and helps regulate blood pressure by inhibiting its rise [33,34].

Chitosan can be used as a polymeric material for microencapsulation [35,36]. Especially, chitosan can be a suitable choice as a polymeric material in microencapsulation using a W/O emulsion system due to its solubility in water [37,38]. Chitosan’s solubility in water varies depending on the content of its amine groups. Therefore, when using chitosan for microencapsulation, its water solubility necessitates a crosslinking process in the final stage of microencapsulation to ensure the stability of the formed microcapsules in water. Glutaraldehyde is commonly used as a crosslinker for this purpose [39,40].

This study was initiated at the request of a small-to-medium-sized cosmetics company based in Andong, South Korea. The company aimed to develop cosmetic ingredients using yam, a major agricultural product of Andong. Accordingly, they sought to stabilize the extract derived from yam through encapsulation, with a preference for using biocompatible materials for the encapsulation process. As a result, chitosan was selected as the encapsulation material in this study. The findings of this research have already been filed as a patent in South Korea, and part of its content has been included in this manuscript.

This study involved extraction using ethanol of active compounds from naturally harvested *Dioscorea quinqueloba*. Two wall materials were prepared to encapsulate the extracts: mucin and chitosan. Mucin was extracted from *Dioscorea quinqueloba*, and chitosan was synthesized from chitin derived from cuttlefish bones. Encapsulation was performed using these two materials in a W/O emulsion system, resulting in microcapsule formation. These microcapsules were evaluated for cytotoxicity, antioxidant effects, and anti-aging properties to assess their potential as cosmetic ingredients.

## 2. Materials and Methods

### 2.1. Reagents and Equipment

The *Dioscorea quinqueloba* used in this study is its scientific name, while in South Korea, it is commonly known as *Danpoongma*. *Dioscorea quinqueloba* was collected from wild mountainous areas in Andong, South Korea. The collection was carried out directly by the authors on 23 September 2024, with the assistance of a specialist experienced in plant identification and harvesting. *Dioscorea quinqueloba* is known to have higher organic acid and saponin contents than other yam varieties [41,42,43]. Freshly harvested *Dioscorea quinqueloba* was stored under refrigeration and used for the study. Samples stored for more than 2 weeks were not used in experiments; instead, newly harvested samples were collected and used for further research. *Dioscorea quinqueloba* were freshly harvested from the soil, thoroughly washed with water to remove any dirt, and then peeled to eliminate the outer skin. Only the remaining white inner flesh was used for the study.

Reagents including ethanol, dimethyl sulfoxide (DMSO), Span 80, mineral oil, diethyl pyrocarbonate, (3-(4,5-dimethylthiazol-2-yl)-2,5-diphenyltetrazolium bromide) (MTT), epigallocatechin gallate (EGCG), TNF-α, glyceraldehyde-3-phosphate dehydrogenase (GAPDH), 2,2-diphenyl-1-picrylhydrazyl (DPPH), butylhydroxytoluene (BHT), L-ascorbic acid (vitamin C), and α-tocopherol (vitamin E) were purchased from Sigma-Aldrich (St. Louis, MO, USA). The TRIzol reagent and TaqMan Universal PCR Master Mix (TUPMM) were purchased from Thermo Fisher Scientific (Waltham, MA, USA).

Scanning electron microscope (SEM) images were obtained using a VEGA 2 LMU instrument (Tescan, Brno, Czech Republic). UV spectra were measured using a Shimadzu UV-1800 spectrophotometer (Kyoto, Japan), and IR spectra were recorded using an IR Spirit-T (Shimadzu, Tokyo, Japan).

### 2.2. Synthesis of Chitosan

Cuttlefish bones were collected and allowed to react with 6 N HCl for 24 h to remove CaCO_3_, followed by reaction with 2 N HCl for 6 h to eliminate CaCO_3_ traces. The remaining material was treated with 4.0% NaOH solution at 100 °C for 12 h to remove proteins, yielding chitin. The obtained chitin was deacetylated with a 50% NaOH solution for 6 h to produce chitosan.

### 2.3. Extraction of Active Compounds from Dioscorea quinqueloba Using Ethanol

*Dioscorea quinqueloba* collected from Andong, Gyeongsangbuk-do, South Korea, was thoroughly washed and peeled.

Prior to extracting the active compounds from *Dioscorea quinqueloba*, the moisture content of the plant material was first determined. To measure the moisture content, *Dioscorea quinqueloba* was sliced into thin sections with a thickness of less than 0.5 cm. The samples were then dried under vacuum conditions at 60 °C for 10 h. The moisture content was calculated based on the difference in weight before and after drying. As a result, the moisture content of *Dioscorea quinqueloba* was found to be 61 ± 3%.

For the extraction of active compounds from *Dioscorea quinqueloba*, the plant material was similarly sliced into thin sections with a thickness of less than 0.5 cm. The cleaned samples were sliced to a 0.5 cm thickness, dried in a hot air oven at 40 °C for 24 h, and ground into a fine powder (100 mesh). The powder (200 g) was extracted with 1 L of ethanol for 7 d. The extract was concentrated under reduced pressure and freeze-dried to obtain 6.4 g of powdered extract. Using ethanol extraction, active compounds were obtained with a yield of 3.2 ± 0.2%.

### 2.4. Extraction of Mucin

*Dioscorea quinqueloba,* collected from Andong, Gyeongsangbuk-do, South Korea, was washed and peeled off. A 200 g sample was blended with 1.0 L of distilled water at 1000 rpm for 1 h. The resulting suspension was then filtered using a cotton cloth to obtain a viscous colloidal solution. After centrifuging at 10 °C for 30 min, precipitates such as starch and saponins were removed. The separation of the precipitate from the colloidal solution was carefully carried out by gently extracting the colloidal solution from the top using a glass dropping pipette. Ethanol (4 times the volume) was added to the remaining colloidal solution, and the mixture was left at 0 °C for 24 h to precipitate mucin. The precipitate was freeze-dried to yield 3.7 g of mucin (yield 1.85%).

### 2.5. Preparation of Microcapsules

To prepare microcapsules, 0.40 g of the synthesized chitosan, 0.20 g of mucin, and 0.060 g of powdered extract obtained from *Dioscorea quinqueloba* were sonicated in 60 mL of water at room temperature for 30 min. Separately, mineral oil (300 mL) and Span 80 (3.0 mL) were mixed. The solution containing chitosan, mucin, and the extracted compound was added dropwise to the mineral oil–Span 80 mixture with stirring at 9000 rpm to form a W/O emulsion. The resulting emulsion was stirred for 20 min using a homogenizer. A saturated toluene solution of glutaraldehyde (3.0 mL) was added for the first crosslinking reaction, followed by stirring for 60 min at 9000 rpm. Then, 1.5 mL of 25% aqueous glutaraldehyde solution was added for the second crosslinking reaction, followed by an additional 60 min of stirring. The formed microcapsules were centrifuged at 5000 rpm for 5 min to separate them from the mineral oil. The residual mineral oil was washed with *n*-hexane and centrifuged at 5000 rpm for 5 min at room temperature. Finally, the microcapsules were vacuum-dried at 30 °C for 24 h. Various parameters, such as the amount of crosslinker, emulsifier, and stirring speed, were varied to evaluate their effects.

The average size of the microcapsules was determined from the SEM images. To do this, 30 microcapsules were selected, excluding those smaller than 200 nm. These extremely small microcapsules were omitted because they are unlikely to function effectively as actual microcapsules and were therefore considered to have no practical value in this context. Additionally, particles with irregular, non-spherical shapes were also excluded. The average size was then calculated based on the selected 30 microcapsules.

### 2.6. Toxicity Test Using MTT Assay

Human foreskin fibroblast Hs68 cells used in this study were purchased from the American Type Culture Collection (ATCC) (Manassas, VA, USA). The cells were cultured in Dulbecco’s modified Eagle’s medium (DMEM) purchased from GenDEPOT (Altair, Troy, MI, USA), supplemented with GenDEPOT 10% fetal bovine serum (FBS) and 1% penicillin–streptomycin (P/S) from Gibco (Billings, MT, USA). Hs68 cells (6 × 10^3^ cells/well) were seeded into 96-well plates and incubated at 37 ± 1 °C and 5% CO_2_ for 24 h. After removing the medium, serum-free DMEM containing the microcapsules was added, and the cells were incubated at 37 ± 1 °C for 24 h. Subsequently, the medium was removed, and 10 µL of MTT solution (5 mg/mL) was added to each well. After 4 h, the medium was removed, and 100 µL of DMSO was added to dissolve the cells. The plates were shaken for 20 min, and the absorbance at 570 nm was measured using a UV spectrophotometer, Shimadzu Corporation, Kyoto, Japan. The cell viability was calculated as follows [44]:

Cell viability (%) = (absorbance of the treated sample/absorbance of the untreated sample) × 100

### 2.7. Antioxidant Effect

The antioxidant activity of the microcapsules was evaluated by measuring their DPPH radical-scavenging activity [45]. The appropriate amount of microcapsules was dissolved in 0.10 mL of ethanol according to the designated concentration and then shaken for 120 min. The solution was mixed with 1.9 mL of 2 × 10^−4^ M DPPH ethanol solution and incubated at 37 °C for 30 min. Absorbance was measured at 516 nm. BHT and vitamins C and E were used as controls. Measurements were performed in triplicate.

### 2.8. Anti-Aging Experiment Using Real-Time Polymerase Chain Reaction (RT-PCR) [46]

Hs68 cells (6 × 10^5^ cells/well) were seeded into 6-well plates and incubated at 37 °C and 5% CO_2_ for 24 h. After stabilization, serum-free DMEM containing the microcapsules was added, and the cells were incubated at 37 °C and 5% CO_2_ for 120 min. TNF-α was added, and the cells were further incubated at 37 °C and 5% CO_2_ for 24 h. Total RNA was extracted using TRIzol reagent and quantified using a NanoDrop (Thermo Fisher Scientific) spectrophotometer. cDNA was synthesized using a ReverTra ACE kit (Toyobo, Osaka, Japan), and RT-PCR was performed using TUPMM, Chongqing Rexon Oil Purification Co., Ltd., Chongqing, China to assess mRNA suppression. Epigallocatechin gallate (EGCG), a bioactive component of green tea, was used as the positive control.

### 2.9. Controlled-Release Experiment [47]

A controlled-release experiment was conducted by adding 0.100 g of microcapsules to 100 mL of 50% ethanol solution in a 100 mL round-bottom flask maintained at 35 °C. The solution was stirred using a magnetic bar, with approximately one rotation every 2 s. Samples (1.0 mL) were collected at predetermined intervals, and the absorbance at 278 nm was measured using a UV spectrophotometer.

## 3. Results and Discussion

In this study, *Dioscorea quinqueloba* collected from the natural environment of Andong was used as the main material. Active compounds were extracted using ethanol, and mucin derived from *Dioscorea quinqueloba* and chitosan derived from cuttlefish bones were used to encapsulate the active compounds to produce microcapsules. The performance of the microcapsules as cosmetic materials was also evaluated.

### 3.1. Chitosan Preparation

Chitin was isolated from cuttlefish bones and deacetylated to produce chitosan. Specifically, the acetamino groups in chitin are converted into amine groups to synthesize chitosan. However, this conversion was incomplete, and residual acetamino groups remained. Therefore, the degree of deacetylation of the prepared chitosan was determined by acid–base titration. The measurements revealed a degree of deacetylation of 93 ± 1%. The molecular weight, determined through a viscosity measurement, was 91,000 ± 2000 g/mol. The IR spectrum of the obtained chitosan is shown in Figure S1 of the Supplementary Materials. The IR spectrum of chitosan obtained in this study closely resembled those reported in previously published literature [48,49], exhibiting no significant discrepancies. These findings indicate that the deacetylation process employed in this study was effectively carried out.

The reason for extracting chitin from cuttlefish bones and synthesizing chitosan in this study is that, while chitin is abundant in nature, the chitin derived from cuttlefish bones is colorless and transparent because it lacks pigments [50]. Consequently, chitosan synthesized from this chitin forms a completely white product. On the other hand, chitin obtained from shrimp shells often retained a reddish pigment.

### 3.2. Extraction of Mucin from Dioscorea quinqueloba

Mucin is a highly viscous glycoprotein characterized by a protein core surrounded by carbohydrates. Because of the challenging separation process, the yield of pure mucin is often very low. In this study, mucin extraction experiments demonstrated significant yield variability across multiple attempts. The IR spectrum of mucin obtained in this study is shown in Figure S2 in the Supplementary Materials.

In the IR spectrum of mucin shown in Figure S2, a strong absorption peak is observed at 3270 cm^−1^, attributed to hydroxy groups, which are part of the carbohydrates on the mucin surface. Additionally, absorptions at 1627 and 1566 cm^−1^ correspond to the amide groups derived from proteins in mucin.

### 3.3. Extraction of Active Compounds from Dioscorea quinqueloba

**Figure 1:**
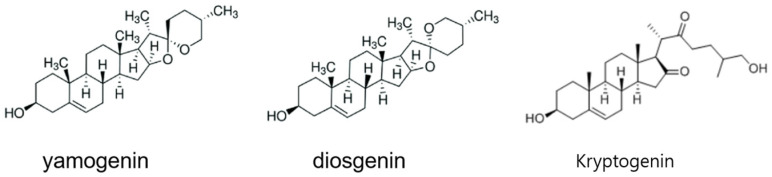
The chemical structures of yamogenin, diosgenin, and kryptogenin.

The extract obtained from *Dioscorea quinqueloba* contains a wide variety of compounds. While the composition of the main components may vary depending on the extraction method, the most well-known bioactive compounds include yamogenin, diosgenin, and kryptogenin [51,52]. Their chemical structures are shown in Figure 1.

The amount of active compounds extracted from *Dioscorea quinqueloba* was examined during extraction. Using ethanol extraction, active compounds were obtained with a yield of 3.2 ± 0.2%. The UV spectrum of the extracted active compounds is shown in Figure S3 of the Supplementary Materials.

### 3.4. Microencapsulation

Microencapsulation of the active compounds extracted from *Dioscorea quinqueloba* was conducted by forming a W/O emulsion using mineral oil as the base. Chitosan and mucin were used as wall materials, and Span 80 served as a surfactant. In the early stages of this study, chitosan was the sole wall material for the microcapsules, and the initial experimental design involved encapsulating mucin together with the extract using chitosan. However, mucin is a high-molecular-weight substance, whereas most of the molecules in the extract have relatively low molecular weights, making it challenging to handle them together. To address this difficulty, the experimental design was modified to first mix mucin with chitosan and then use this mixture for encapsulating the extract.

**Figure 2:**
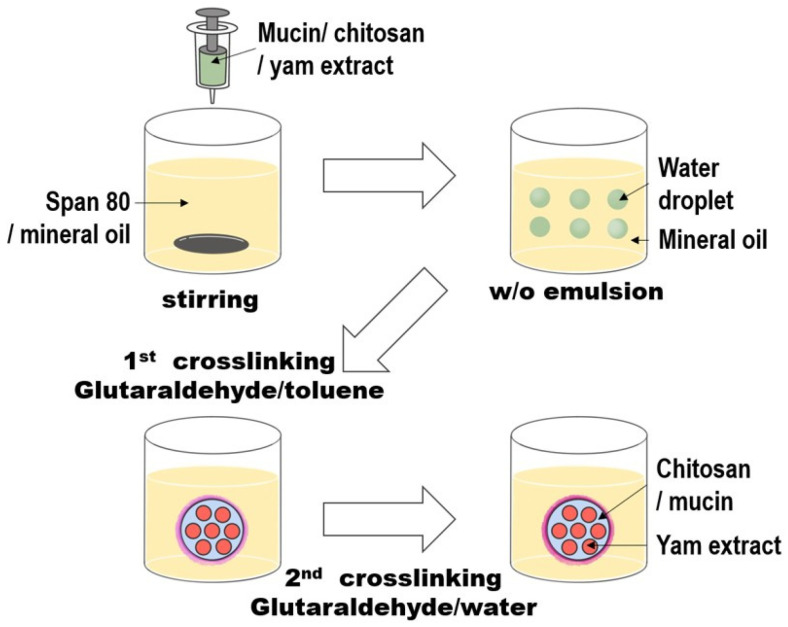
Microcapsule preparation process.

Glutaraldehyde was used as a crosslinking agent. A single glutaraldehyde molecule reacts with two amine groups of chitosan to form imine groups, facilitating crosslinking [39,40]. The overall microencapsulation process is illustrated in Figure 2.

**Figure 3:**
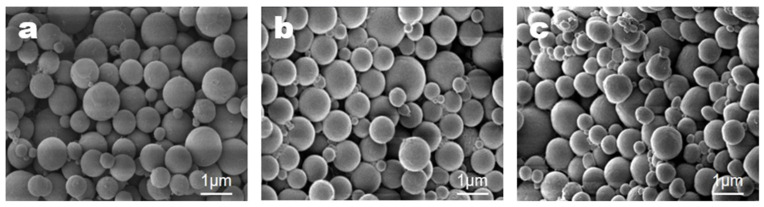
SEM images of microcapsules formed at different stirring speeds: (**a**) 6000, (**b**) 9000, and (**c**) 12,000 rpm.

Scanning electron microscope (SEM) images were obtained and analyzed to examine the microcapsule particle morphology. SEM images were obtained using a VEGA 2 LMU instrument (Tescan, Brno, Czech Republic). The effects of varying experimental conditions on microcapsule formation were investigated. First, the effect of the stirring speed on microcapsule formation was studied. All other conditions were maintained as described in the representative method, and the stirring speed was varied between 6000, 9000, and 12,000 rpm. The results are shown in Figure 3.

As shown in Figure 3, the size of the microcapsules decreased as the stirring speed increased. The average sizes were calculated as follows: 1130 ± 120 nm at 6000 rpm, 920 ± 80 nm at 9000 rpm, and 890 ± 80 nm at 12,000 rpm. These results indicate that increasing the stirring speed reduced the size of the microcapsules. In particular, the microcapsules formed at 9000 and 12,000 rpm were almost identical in size. Additionally, the size and shape distributions of the microcapsules were nearly uniform, leading to the selection of 9000 rpm as the standard stirring speed for the representative method.

**Figure 4:**
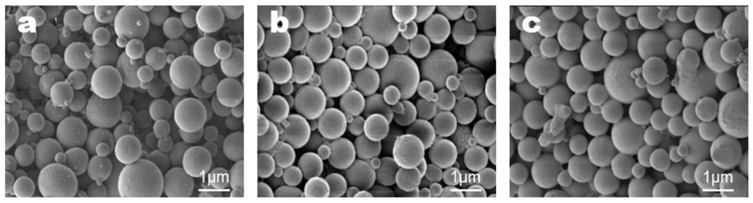
SEM images of microcapsules formed at different emulsifier concentrations: (**a**) 0.5, (**b**) 1.0, and (**c**) 2.0%.

The second condition investigated was the emulsifier concentration. Microcapsules were prepared with emulsifier concentrations of 0.5, 1.0, and 2.0%, and all other conditions were maintained as described in the representative method. The results are shown in Figure 4.

From the results shown in Figure 4, the average microcapsule sizes formed at each emulsifier concentration were 1090 ± 100 nm at 0.5%, 920 ± 80 nm at 1.0%, and 900 ± 80 nm at 2.0%. Increasing the emulsifier concentration reduced the average size of the microcapsules. At 0.5%, the microcapsules were relatively less uniform than those formed at 1.0 and 2.0% and exhibited similar sizes and appearances. Based on these observations, an emulsifier concentration of 1.0% was selected as representative.

The third condition investigated was the concentration of glutaraldehyde used as the crosslinking agent. Under the same conditions described in the representative method, microcapsules were prepared by varying the amount of glutaraldehyde-saturated toluene solution added during the first cross-linking step (1.5, 3.0, and 4.5 mL). Similarly, during the second crosslinking step, a 25% aqueous glutaraldehyde solution was added in varying amounts (1.0, 1.5, and 2.0 mL). The SEM analysis of the microcapsules formed under these six conditions revealed no significant morphological differences. Therefore, it can be concluded that variations in the amount of crosslinking agent have little to no effect on the morphology of the formed microcapsules.

### 3.5. Toxicity Test

**Figure 5:**
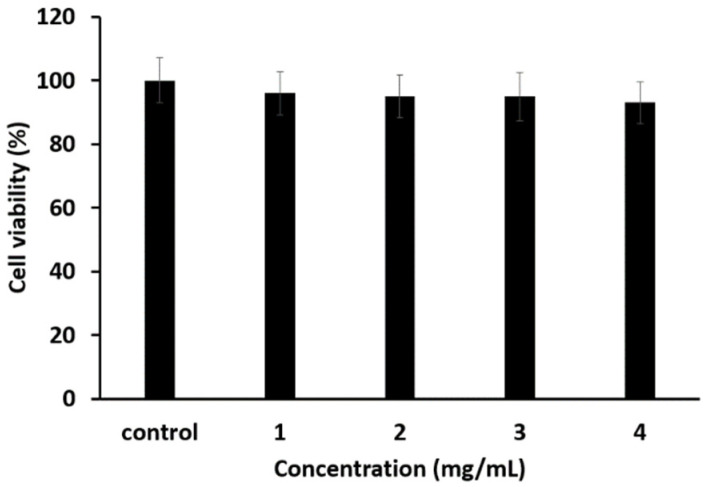
Microcapsule toxicity test using MTT assay.

A toxicity test for the microcapsules prepared using the representative method was conducted using an MTT assay. The results are shown in Figure 5.

The results shown in Figure 5 indicate that the microcapsules did not exhibit toxicity, even at a concentration of 4 mg/mL.

### 3.6. Antioxidant Effect

**Figure 6:**
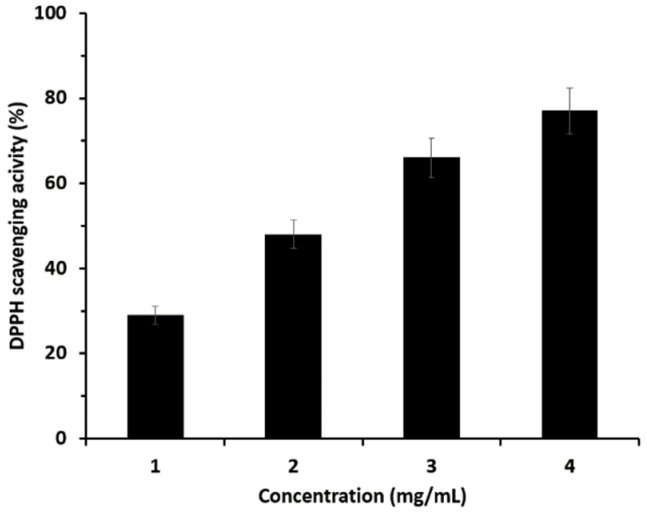
Antioxidant effect based on the concentration of microcapsules.

The antioxidant effect was measured by DPPH scavenging activity. The results of the experiment conducted using microcapsules prepared using a representative method are shown in Figure 6.

**Figure 7:**
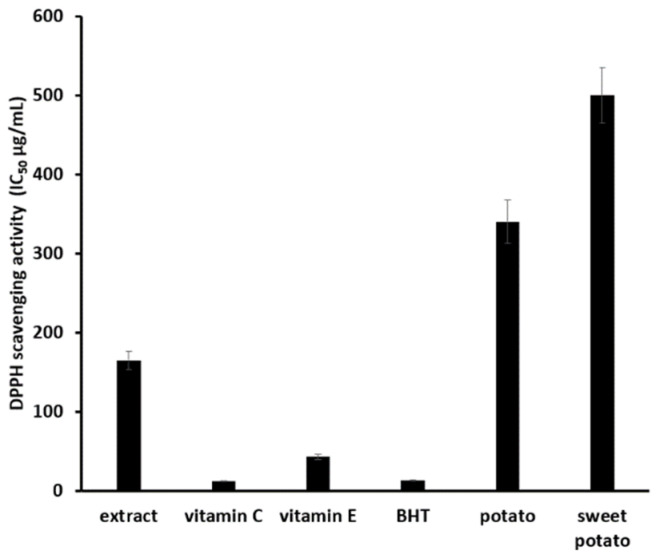
Comparative experiment on the antioxidant effects of extracts.

The experimental results shown in Figure 6 indicate that the antioxidant capacity of the microcapsules increased continuously as the concentration increased from 1 to 4 mg/mL. To assess the relative strength of this antioxidant activity, comparisons were made with well-known antioxidants such as vitamin C, BHT, and vitamin E, as well as potato and sweet potato extracts, which have moderate antioxidant properties. Potato and sweet potato extracts were prepared using the same extraction process as described in this study. The antioxidant effects were expressed as IC50, the concentration required to scavenge 50% of the DPPH radicals. The results are shown in Figure 7.

The results shown in Figure 7 indicate that while it is difficult to compare the antioxidant ability of the microcapsules with that of exceptional antioxidants such as vitamin C, BHT, and vitamin E, the microcapsules still demonstrate significant antioxidant capacity. The antioxidant ability of the microcapsules was approximately twice that of potato extract and three times that of sweet potato extract, both known to have moderate antioxidant properties.

### 3.7. Anti-Aging Effect

**Figure 8:**
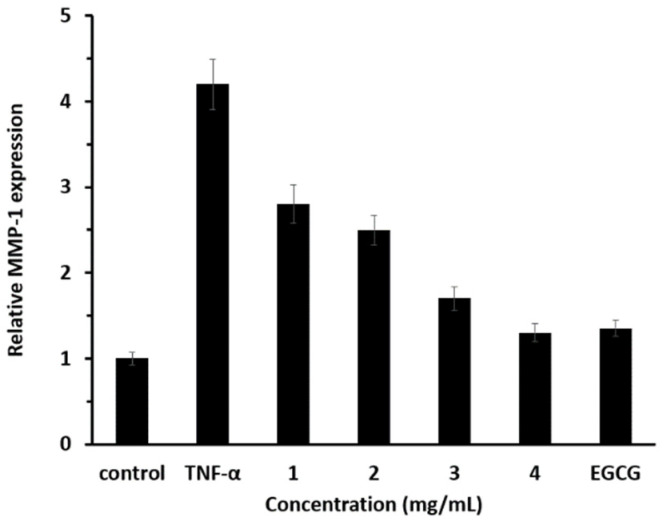
Anti-aging effect of microcapsules.

In this study, the anti-aging effects were measured by a real-time polymerase chain reaction (RT-PCR). The results are presented in Figure 8. The concentration of EGCG used was 100 μg/mL, comparable to the concentration of the extract released from 4 mg/mL of microcapsules.

The anti-aging effect was evaluated based on the relative expression of MMP-1 (Figure 8). When the microcapsule concentration was 4 mg/mL, the amount of extract released was approximately 100 μg/mL, which is within the margin of error or slightly lower than this value. This enabled a direct comparison of the effects of microcapsules with those of EGCG. The fact that the results of these two cases were very similar indicates that the microcapsules exhibited an anti-aging effect comparable to that of EGCG.

### 3.8. Controlled Release

**Figure 9:**
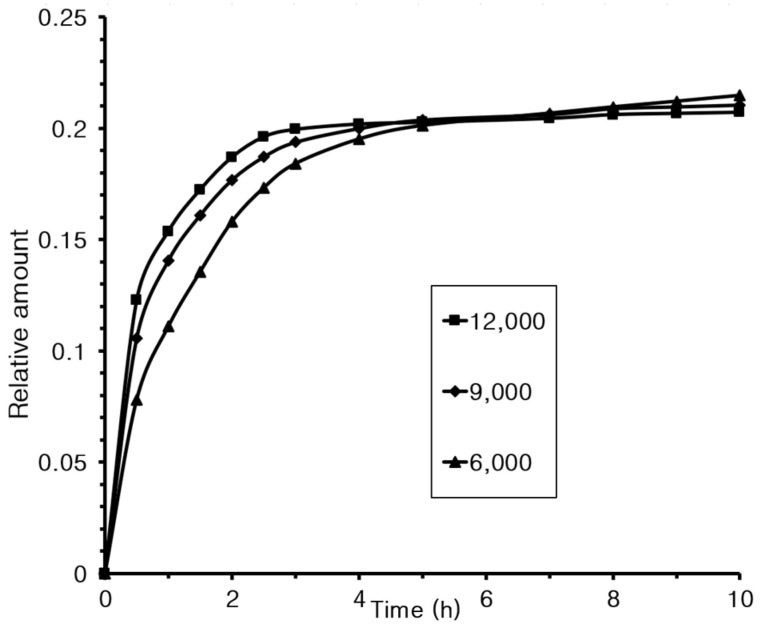
Release experiment results at different stirring speeds.

Controlled-release experiments were conducted under conditions identical to those used in the representative method, except the stirring speed varied. The results are presented in Figure 9.

The results shown in Figure 9 indicate that increasing the stirring speed accelerated the microcapsule release rate. This is believed to be related to the size of the microcapsules formed. Specifically, the average sizes were 1130 ± 120 nm at 6000 rpm, 920 ± 80 nm at 9000 rpm, and 890 ± 80 nm at 12,000 rpm.

**Figure 10:**
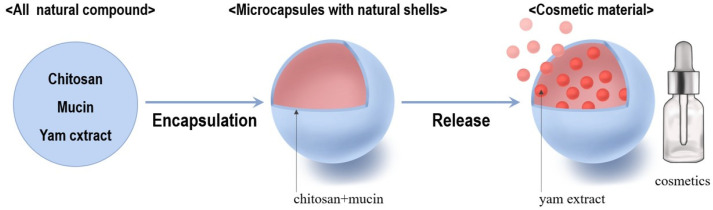
Formation of microcapsules from yam extract and their application in cosmetics.

In conclusion, this study successfully extracted a bioactive extract from *Dioscorea quinqueloba*. The extract was then encapsulated using natural materials, chitosan and mucin, to create microcapsules that can be utilized as a fundamental ingredient in cosmetics. The entire process is illustrated in Figure 10.

## 4. Conclusions

In this study, active compounds were extracted using ethanol from *Dioscorea quinqueloba* collected in Andong. These extracts were encapsulated in mucin extracted from the same plant and chitosan prepared from cuttlefish. Encapsulation was carried out in a W/O emulsion system and completed in two crosslinking stages. SEM analysis confirmed that the microcapsules were spherical, with sizes ranging from 890 to 1130 nm, depending on the manufacturing conditions. The resulting microcapsules were evaluated as cosmetic materials by measuring their toxicity, antioxidant properties, and anti-aging effects. The results demonstrate that the microcapsules exhibited no toxicity, had significantly superior antioxidant properties compared to potato and sweet potato extracts, and had excellent anti-aging effects.

## Data Availability

The original contributions presented in this study are included in the article/Supplementary Materials. Further inquiries can be directed to the corresponding author.

## Supplementary Materials

The following supporting information can be downloaded at: https://www.mdpi.com/article/10.3390/ma18102178/s1, Figure S1: IR spectrum of synthesized chitosan. Figure S2: IR spectrum of extracted mucin. Figure S3: UV spectrum of extracted active compounds.
