# Supplementary material for: Extraction of Active Compounds from Dioscorea quinqueloba and Their Encapsulation Using Mucin and Chitosan for Application in Cosmetic Formulations
Source: Materials (Basel). 2025 May 8;18(10):2178. doi: 10.3390/ma18102178 (PMC12113435; doi:10.3390/ma18102178)
Supplement: Supplementary file 1 [file materials-18-02178-s001.zip › materials-3504304-supplementary.pdf]

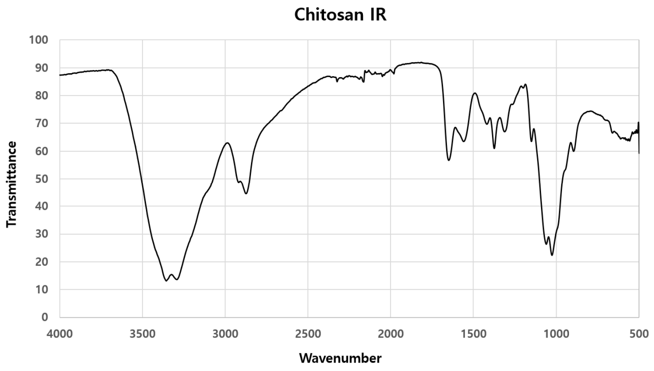

Figure S1. IR spectrum of synthesized chitosan.

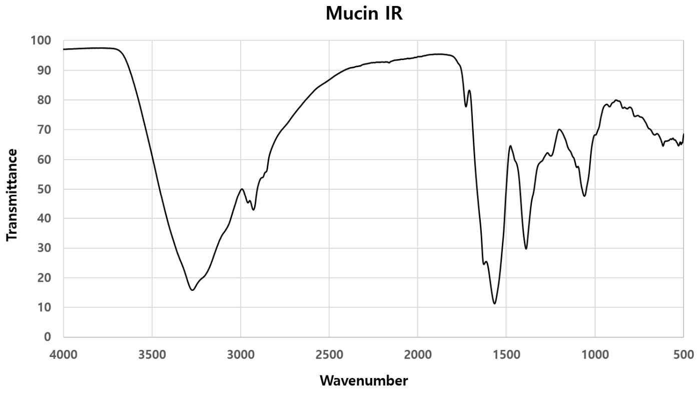

Figure S2. IR Spectrum of extracted mucin.

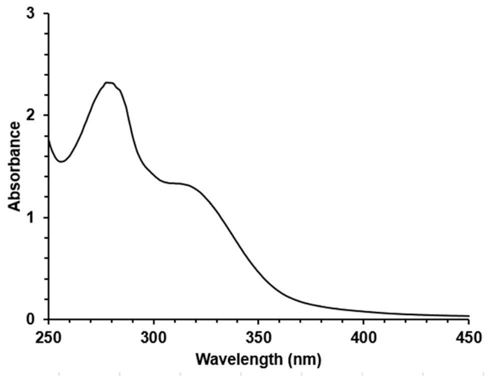

Figure S3. UV spectrum of extracted active compounds
